# Knowledge, Attitudes and Practices of Animal Bite Victims Attending an Anti-rabies Health Center in Jimma Town, Ethiopia

**DOI:** 10.1371/journal.pntd.0003867

**Published:** 2015-06-26

**Authors:** Tadele Kabeta, Benti Deresa, Worku Tigre, Michael P. Ward, Siobhan M. Mor

**Affiliations:** 1 Jimma University College of Agriculture and Veterinary Medicine, Jimma, Ethiopia; 2 School of Veterinary Medicine, Wollega University College of Medical and Health Sciences, Nekemte, Ethiopia; 3 Faculty of Veterinary Science, The University of Sydney, Sydney, New South Wales, Australia; 4 Marie Bashir Institute for Infectious Diseases and Biosecurity, The University of Sydney, Sydney, New South Wales, Australia; Swiss Tropical and Public Health Institute, SWITZERLAND

## Abstract

**Background:**

Rabies is an important but preventable cause of death in Ethiopia. We assessed the knowledge, attitudes and practices of animal bite victims attending an anti-rabies health center in Jimma Town, Ethiopia.

**Methodology/Principal Findings:**

Between July 2012 and March 2013 a cross-sectional questionnaire was administered to 384 bite victims or their guardians in the case of minors (aged <15 years). Factors associated with knowledge, attitudes and practices were evaluated using generalized linear models. Almost all participants (99%) were aware that rabies was transmitted by the bite or lick of a rabid dog, however only 20.1% identified “germs” as the cause of disease. A majority of participants stated rabies could be prevented by avoiding dog bites (64.6%) and confining dogs (53.9%); fewer (41.7%) recognized vaccination of dogs/cats as an important preventive strategy. Regarding attitudes, most (91.1%) agreed that medical evaluation should be sought as soon as possible. However, most (75.0%) also believed that traditional healers could cure rabies. Rural residence (adjusted odds ratio [OR] = 2.1, p = 0.015) and Protestant religion (OR = 2.4, p = 0.041) were independently associated with this belief. Among 186 participants who owned dogs, only 9 (4.8%) had ever vaccinated their dog and more than 90% of respondents indicated that their dog was free-roaming or cohabitated with the family. Only 7.0% of participants applied correct first aid following exposure, and the majority (47.7%) reported that the animal was killed by the community following the incident. Female sex and Muslim religion were independently associated with higher and lower practices scores, respectively, due largely to differences in animal management practices following the incident.

**Conclusions/Significance:**

Although respondents demonstrated reasonably sound knowledge of rabies and its transmission, attitudes and practices were inconsistent with rabies prevention. Culturally- and gender-sensitive activities that promote proper first aid and healthcare seeking behavior as well as appropriate animal management, particularly in rural areas, are needed to prevent deaths associated with rabies in this setting.

## Author Summary

Rabies is an important but preventable cause of death in Ethiopia. We assessed the knowledge, attitudes and practices of animal bite victims attending an anti-rabies health center in Jimma Town, Ethiopia. We found generally high levels of knowledge about rabies. Participants recognized domestic dogs as the source and identified a range of appropriate preventive measures, including avoidance of bites and the need for dog confinement. Despite this reasonable level of knowledge, attitudes and practices that were not consistent with rabies prevention were identified. In particular, a belief that rabies could be cured by traditional healers (herbal medicine) was common among the participants, especially among those who lived in rural areas or who were of Protestant faith. Further, few of the dog owners actually confined their dogs and practiced vaccination. First aid following a suspect rabid bite was inadequate in the majority of participants and, contrary to established guidelines, most animals were killed rather than placed under quarantine following a bite incident. Female bite victims were more likely to report improved animal management practices while those of Muslim faith tended to report unfavorable animal management practices. These findings highlight the need for culturally- and gender-sensitive awareness raising programs that improve first aid and healthcare seeking behavior as well as appropriate animal management in order to prevent rabies-related deaths in Jimma Zone, Ethiopia.

## Introduction

As a disease that mostly impacts poor communities, rabies is a classic example of a neglected tropical disease [1]. A vaccine-preventable disease, most deaths from rabies arise due to lack of awareness and poor access to proper health services [2]. It is estimated that around half of the global human population lives in canine rabies-endemic countries and is at risk of exposure [3]. Because cases often go unreported, it is agreed that official records vastly under-estimate the true burden of rabies [3–6]. Modelling studies estimate the annual human death toll from rabies to be around 50,000–60,000, with 99% of these fatalities occurring in tropical developing countries, overwhelmingly in Africa and Asia [3,6–8]. An estimated 21,000–24,000 these deaths occur annually in Africa alone [6,8]. More recently the global burden of rabies (reflecting the number of human deaths as well as lost productivity due to premature death, adverse events related to the nerve-tissue vaccine and psychological effects of the disease) was estimated at 3.7 million disability-adjusted life years (DALYs), while global economic losses associated with the disease were estimated at 8.6 billion USD annually [8].

Rabies is spread principally by domestic dogs [9,10] and these animals constitute the major source of infection to humans [6]. In Ethiopia, for example, dogs were the source of infection in 95% of fatal human cases between 1990 and 2000 [11]. Effective vaccines are available to control disease in dogs and large-scale elimination of canine rabies is considered epidemiologically and logistically feasible as well as cost-effective [12,13]. Despite effective tools and sound economic rationale for control, rabies remains a neglected disease in many parts of Africa [12,14].

Rabies has long been recognized as an important disease in Ethiopia [15]. Several studies have investigated rabies awareness among urban [16,17] and rural [18–21] communities in Ethiopia, however none have investigated knowledge, attitudes and practices among bite victims, specifically. One study, conducted in North Gondar Zone (95% Ethiopian Orthodox religion [22]) in northern Ethiopia, found high awareness of the disease but a dependence on traditional healers for treatment [18]. Ethiopia has a diverse population and thus knowledge, attitudes and practices might be expected to differ by geographic region. We assessed the knowledge, attitudes and practices of animal bite victims visiting an anti-rabies health center in Jimma Zone (86% Muslim religion [22]), which differs substantially from North Gondor in terms of religious composition.

## Materials and Methods

### Study area

Jimma Town Anti-rabies Health Center (JTAHC) is located in Jimma Town, the capital of Jimma Zone in Oromia National Region, Ethiopia. Jimma Town (7°13’ to 8°56’ N, 35°52’ to 37°37’E) is 352 km southwest of Addis Ababa. The Zone lies at an elevation of 880 to 3360 meters above sea level, and experiences hot and humid weather conditions. JTAHC provides a range of health services to the population of Jimma Zone (approximately 2.5 million people in 2007 [22]) and is the only center providing post-exposure prophylaxis (PEP) for humans bitten by animals suspected of rabies within this zone and surrounding areas.

### Study population and methods

The target population for this cross-sectional survey was animal bite victims or their guardians presenting for the first time to JTAHC between July 20, 2012 and March 12, 2013. This research was approved by the research and ethical review committee of the College of Agriculture and Veterinary Medicine of Jimma University. After explaining the study, written consent was obtained from all patients or their guardians (in case of minors aged <15 years, per cultural norms in Ethiopia).

A closed and open-ended questionnaire was developed and included questions to explore participant knowledge of rabies (including means of transmission to humans and its treatment); their attitudes towards the public health risk of rabies; and their practices for prevention and control of the disease. Practices related to seeking PEP were not assessed because the study specifically targeted bite victims presenting to the health center to receive PEP. The questionnaire also collected basic demographic information, including gender, age, educational status (no formal education, 1–8 (primary), 9–12 (secondary) and >12 (tertiary)), and religion (Orthodox, Muslim and Protestant), residence (urban and rural) and occupation (farmers, students and working professionals). The questionnaire was translated into local languages (Afan Oromo and Amharic) and then back-translated to English and pre-tested to ensure validity.

### Statistical analysis

The statistical approach was adapted from Davlin and colleagues [23]. Briefly, for each of the question types (knowledge, attitudes, practices) responses were designated as “favorable” or “unfavorable” and compiled into a score based on the number of “favorable” responses. A favorable responses was defined as a response that is consistent with rabies prevention. For example, knowledge that rabies is transmitted by dog bites was considered “favorable” since people with this knowledge can prevent rabies through avoiding dog bites and/or seeking medical care following dog bite. To reduce the influence of the large number of clinical signs relative to other parameters on the knowledge score, we assigned +1, +2 or +3 to respondents who identified below the 25th percentile, between the 25th and 75th percentile and above the 75th percentile, respectively, for this question. Potential factors associated with knowledge, attitudes and practice scores were identified in univariate analysis using t-tests and ANOVA, or the non-parametric equivalent, as appropriate. Comparisons yielding a p-value of <0.1 were included in subsequent multivariate analysis. Multivariate, Poisson regression was employed to model scores based on potential factors identified in the univariate analysis. In addition, chi square analysis was employed to examine factors specifically associated with a belief that traditional healers and herbal medicines can cure rabies. Factors associated with this belief were included in subsequent multivariate, logistic regression. All models were fitted using the glm function in SPSS, version 21 (IBM Corporation, Armonk, NY).

## Results

### Participant demographics and circumstances surrounding exposure

Table 1 shows the demographic characteristics of 384 respondents who were interviewed as part of the study. Of these, 254 (66.1%) respondents presented to the health center because they themselves had been potentially exposed to rabies. Remaining respondents were parents (25.3%) or older siblings (8.6%) who brought a minor (<15 years) to the health center for treatment following potential exposure. Cases presented to the health center within 3 days (50.5%), 4–7 days (39.8%) or more than 8 days (9.7%) following potential exposure. Eighty-three per cent of potential exposures were inflicted by dogs; other sources included cats (6.8%), bovines (3.4%), equines (2.6%), wildlife (specifically, foxes and hyenas; 0.8%) and humans (3.4%). In the 319 cases where a dog was the source of potential exposure, respondents indicated the animal was owned by the family (25.1%), owned by a neighbor (45.8%) or free-roaming (29.2%). Bites were the main type of exposure (82.6%). Remaining cases sought treatment following contact with broken skin (12.5%), scratches without bleeding (1.3%) or non-specific contact with an animal or human suspected of having rabies (3.6%). Sites of exposure were the head/face (4.2%), chest (7.6%), arm/hand (26.6%) and leg (61.7%).

**Table 1:** Socio-demographic characteristics of participants in a study of rabies knowledge, attitudes and practices in Jimma Town, Ethiopia, 2012–2013 (n = 384).

Characteristic	No. (%)
Age of respondent	
>30 years	156 (40.6)
≤30 years	228 (59.4)
Sex of respondent	
Female	120 (31.3)
Male	264 (68.8)
Religion	
Protestant	54 (14.1)
Muslim	164 (42.7)
Orthodox	166 (43.2)
Educational status	
Tertiary	29 (7.6)
Secondary	40 (10.4)
Primary	197 (51.3)
No formal education	118 (30.7)
Type of employment	
Farmer	214 (55.7)
Student	71 (18.5)
Working professional	99 (25.8)
Residence	
Rural	229 (77.8)
Urban	85 (22.1)
Dog ownership	
Yes	186 (48.4)
No	198 (51.6)
Know someone who has been exposed to rabies or a suspect rabid animal	
Yes	197 (51.3)
No	187 (48.7)

### Knowledge

Table 2 shows the knowledge of participants in relation to rabies transmission and prevention. The vast majority (91.7%) of participants stated that they had heard of rabies before they had been exposed. More than 60% of respondents indicated they heard about the disease from family; friends (21.1%), teachers (10.7%) and mass media (5.5%) were also sources of information. Despite the high awareness, most respondents (52.3%) did not know what caused the disease and more than 90% stated that the maximum incubation period was less than 40 days in humans. Most participants were able to identify the main clinical signs of rabies in animals and humans, although hydrophobia was less commonly identified (56.8% and 63.8% respondents recognized this as a sign in animals and humans, respectively). Almost all participants (99.0%) identified a bite from a rabid dog as the main way that people acquire rabies, with fewer participants appreciating the role of rabid cats (76.3%), farm animals (71.1%) and wildlife (47.7%) in transmission. More than a third of participants stated that meat and milk of rabid animals could transmit the disease to humans.

**Table 2:** Knowledge of participants in a study of rabies knowledge, attitudes and practices in Jimma Town, Ethiopia, 2012–2013 (n = 384).

Knowledge parameter	No. (%)
*Awareness of disease agent*	
Know that rabies is caused by a germ	
Yes [+1]	77 (20.1)
No	106 (27.6)
Don’t know	201 (52.3)
*Awareness of rabies transmission*	
Bite or lick from rabid dog	
Yes [+1]	380 (99.0)
No	4 (1.0)
Bite from other^1^ rabid animal	
3 other [+3]	159 (41.1)
2 other [+2]	92 (24.0)
1 other [+1]	91 (23.7)
None	43 (11.2)
Don’t know	31 (8.1)
*Awareness of clinical presentation*	
Number of correct clinical signs in animals, median (IQR)	5 (2.0)
Number of correct clinical signs in humans, median (IQR)	5 (1.0)
Maximum incubation period in humans	
>40 days [+1]	0
<40 days	356 (92.7)
Don’t know	25 (7.3)
*Awareness of rabies prevention*	
Aware that person should go to health facility if bitten by animal suspected of having rabies	
Yes [+1]	324 (84.4)
No^2^	60 (15.6)
Avoid dog bite	
Yes [+1]	248 (64.6)
No	136 (35.4)
Vaccinate dogs/cats	
Yes [+1]	160 (41.7)
No	224 (58.3)
Eliminating strays or free roaming dogs	
Yes [+1]	278 (72.4)
No	106 (27.6)
Confining dogs	
Yes [+1]	207 (53.9)
No	177 (46.1)

Scores assigned to “favorable” responses are indicated in square parentheses.

^1^ Includes cats (n = 293), domestic livestock (273) and wild canines (183).

^2^ Alternatives include traditional healer (n = 58), spiritual healer (1), and nowhere (1).

### Attitudes

Participant attitudes towards rabies are shown in Table 3. Around 75% of respondents believed that traditional healers and herbal medicines cure rabies. In open-ended questions, bite victims from remote rural areas mentioned that they came to the health center due to their past experience of observing clinical disease and deaths among a neighbor, family member or close relative who was taken to a traditional healer. Others mentioned that they came to the health center because they could not comply with instructions provided by a traditional healer. For example, farmers that owned lands spanning two sides of a river could not comply with advice that they not cross a river within 40 days of receiving a herbal remedy. Among 309 respondents who had some experience with PEP by the time of the interview, 134 (43.3%) indicated that multiple injections were tolerable, 175 (56.6%) indicated that it was difficult to finish all the doses, and 24 (7.8%) stated that they prefer traditional medicines.

**Table 3:** Attitudes of participants in a study of rabies knowledge, attitudes and practices in Jimma Town, Ethiopia, 2012–2013 (n = 384).

Attitude parameter	No. (%)
Thinks rabies is a health risk to them	
Agree/strongly agree [+1]	384 (100.0)
Disagree/strongly disagree	0
Thinks individuals exposed to rabies should seek medical evaluation as soon as possible	
Agree/strongly agree [+1]	367 (91.1)
Disagree/strongly disagree	17 (8.9)
Thinks traditional healers and herbal medicine cures rabies	
Agree/strongly agree	291 (75.8)
Disagree/strongly disagree [+1]	93 (24.2)
Thinks rabies is preventable	
Agree/strongly agree [+1]	288 (75.0)
Disagree/strongly disagree	96 (25.0)
Thinks keeping free roaming/unvaccinated dogs is ok	
Agree/strongly agree	28 (7.3)
Disagree/strongly disagree [+1]	356 (92.7)
Thinks it is impossible to live without pets	
Agree/strongly agree	99 (25.8)
Disagree/strongly disagree [+1]	285 (74.2)

Scores assigned to “favorable” responses are indicated in square parentheses and reflect attitudes consistent with rabies prevention

### Practices

Table 4 shows the practices adopted following potential exposure to rabies. In 62 cases the animal was deemed to have died of the disease while under observation. Among 186 participants who owned dogs, only 9 (4.8%) reported that they vaccinated their dog. More than 90% of respondents indicated that their dog was free-roaming or cohabitated with the family; only 16 (8.6%) reported that the pet was confined. If a ruminant was suspected of having rabies, 133 (34.6%) respondents indicated that they would slaughter and eat the animal, while 251 (65.4%) stated that the animal would be killed and burned.

**Table 4:** Practices of participants in a study of rabies knowledge, attitudes and practices in Jimma Town, Ethiopia, 2012–2013 (n = 384).

Practice parameter	No. (%)
*Circumstances following bite incident*	
First aid applied following bite by suspected rabid animal	
Wash wound vigorously with soap, iodine [+1]	27 (7.0)
Wash wound vigorously with water	192 (50.0)
Nothing	165 (43.0)
Status of animal following bite incident	
Animal under quarantine/died under observation [+1]	138 (35.9)
Killed by community	183 (47.7)
Don’t know	63 (16.4)

Scores assigned to “favorable” responses are indicated in square parentheses and reflect practices consistent with rabies prevention.

### Factors associated with knowledge, attitudes and practices

Factors associated with knowledge, attitudes and practices scores are shown in Table 5. Rural residence was the only significant factor associated with attitudes score in univariate analysis, while age, sex and religion were significantly associated with practices score. Female sex (crude β = 0.358, p = 0.035; adjusted β = 0.330, p = 0.039) and Muslim religion (crude β = -0.442, p = 0.011; adjusted β = -0.413, p = 0.018) remained significant in multivariate analysis and were associated with higher and lower practices scores, respectively. There was no significant interaction between gender and religion. The proportion of women reporting that the animal was killed was 38.3% (vs. 45.8% of men, p = 0.015) while 59.1% of Muslims reported that the animal was killed (vs. 39.1% of other religions, p<0.001). Table 6 shows the factors associated with a belief that traditional healers and herbal medicines cures rabies. Rural residence (odds ratio [OR] = 2.1, p = 0.015) and Protestant religion (OR = 2.4, p = 0.041) were independently associated with this belief. There was no significant interaction between place of residence and religion.

**Table 5:** Scores of participants in a study of rabies knowledge, attitudes and practices in Jimma Town, Ethiopia, 2012–2013 (n = 384).

Factor	Knowledge score (out of 17)		Attitudes score (out of 6)		Practices score (out of 2)	
	Mean (SD)	p-value	Mean (SD)	p-value	Mean (SD)	p-value
Age of respondent						
>30 years	9.8 (2.0)	0.259	4.6 (0.84)	0.234	0.4 (0.54)	0.041
≤30 years	9.5 (1.9)		4.7 (0.78)		0.5 (0.58)	
Sex of respondent						
Female	9.6 (1.9)	0.899	4.7 (0.76)	0.593	0.5 (0.58)	0.009
Male	9.6 (2.0)		4.6 (0.83)		0.4 (0.56)	
Religion						
Protestant	9.5 (2.1)	0.332	4.6 (0.74)	0.932	0.5 (0.57)	0.011
Muslim	9.5 (1.9)		4.6 (0.76)		0.3 (0.53)	
Orthodox	9.8 (2.0)		4.6 (0.88)		0.5 (0.59)	
Educational status						
Tertiary	10.4 (2.1)	0.105	4.7 (0.80)	0.755	0.3 (0.54)	0.211
Secondary	9.3 (1.9)		4.5 (0.88)		0.4 (0.54)	
Primary	9.6 (1.8)		4.6 (0.84)		0.5 (0.59)	
No formal education	9.7 (2.1)		4.7 (0.73)		0.4 (0.55)	
Type of employment						
Farmer	9.7 (1.9)	0.654	4.6 (0.79)	0.171	0.4 (0.56)	0.442
Student	9.5 (1.8)		4.5 (0.87)		0.5 (0.61)	
Working professional	9.5 (2.1)		4.7 (0.80)		0.4 (0.55)	
Residence						
Rural	9.6 (1.9)	0.427	4.6 (0.81)	0.008	0.4 (0.57)	0.586
Urban	9.7 (2.1)		4.8 (0.78)		0.4 (0.56)	
Dog ownership						
Yes	9.6 (1.9)	0.888	4.6 (0.87)	0.726	0.4 (0.54)	0.847
No	9.6 (2.0)		4.6 (0.74)		0.4 (0.59)	
Know someone who has been exposed to rabies or a suspect rabid animal						
Yes	9.8 (1.9)	0.084	4.6 (0.80)	0.407	0.4 (0.55)	0.768
No	9.4 (2.0)		4.7 (0.81)		0.4 (0.59)	

**Table 6:** Factors associated with a belief that traditional healers and herbal medicine cures rabies, in a study of rabies knowledge, attitudes and practices in Jimma Town, Ethiopia, 2012–2013 (n = 384).

Factor	No. (%)	Univariate analysis		Multivariate analysis	
		Crude OR (95% CI)	p-value	Adjusted OR (95% CI)	p-value
Age of respondent					
>30 years	123 (0.79)	1.33 (0.82, 2.16)	0.246	1.30 (0.76, 2.22)	0.338
≤30 years	168 (0.74)	1.0		1.0	
Sex of respondent					
Female	85 (0.71)	0.68 (0.42, 1.12)	0.127		
Male	206 (0.78)	1.0			
Religion					
Protestant	46 (0.85)	2.34 (1.03, 5.32)	0.039	2.43 (1.04, 5.69)	0.041
Muslim	127 (0.77)	1.40 (0.85, 2.30)	0.187	1.20 (0.70, 2.06)	0.505
Orthodox	118 (0.71)	1.0		1.0	
Educational status					
Tertiary	17 (0.59)	0.38 (0.16, 0.90)	0.025	0.58 (0.22, 1.52)	0.265
Secondary	30 (0.75)	0.80 (0.35, 1.87)	0.616	1.10 (0.44, 2.71)	0.843
Primary	151 (0.77)	0.88 (0.51, 1.53)	0.656	0.89 (0.48, 1.67)	0.726
No formal education	93 (0.79)	1.0		1.0	
Type of employment					
Farmer	174 (0.81)	2.37 (1.39, 4.07)	0.001	1.39 (0.70,2.77	0.344
Student	53 (0.75)	1.61 (0.82, 3.16)	0.165	1.15 (0.53, 2.51)	0.721
Working professional	64 (0.65)	1.0			
Residence					
Rural	240 (0.80)	2.71 (1.61, 4.56)	<0.001	2.12 (1.16, 3.88)	0.015
Urban	51 (0.60)	1.0		1.0	
Pet ownership					
Yes	138 (0.74)	0.85 (0.53, 1.35)	0.482		
No	153 (0.77)	1.0			
Know someone who has been exposed to rabies or a suspect rabid animal					
Yes	158 (0.80)	1.64 (1.03, 2.64)	0.038	1.55 (0.94, 2.54)	0.085
No	133 (0.71)	1.0		1.0	

## Discussion

This study is the first to assess the knowledge, attitudes and practices among attendees at an anti-rabies health center in Jimma Town, Ethiopia. Similar to other studies conducted elsewhere in Ethiopia [16,18,21], we found moderately high levels of knowledge regarding the role of dogs in transmission as well as clinical signs in both animals and humans. However, unfavorable attitudes and practices were found in terms of the need for appropriate first aid, medical care and animal management.

Of the 384 respondents, 91.7% had heard of rabies before exposure. This suggests that victims are aware of the presence of rabies in the area and likely reflects the endemicity of the disease in this location. The majority (83.9%) of the victims had heard about rabies from their family or friends. This finding is similar to the work of Sambo and colleagues who reported the most common source of information on rabies in Tanzania was from personal contacts (neighbors, parents and friends) [24]. Only 16.2% of respondents in this study had heard about rabies from sources such as media and teachers. This suggests that dissemination of information from the government is poor, and could be due to the fact that rabies is neglected and not considered a major public health importance in Jimma Zone.

Most of the respondents were able to identify the typical clinical signs of rabies for both animals and humans and were aware that rabies is transmitted via bites or licks from rabid dogs, cats and other domestic animals. This is consistent with previous reports from Ethiopia [16,17] and other countries [25,26] and again suggests that the disease has long been recognized in Jimma Town and surrounding areas. While awareness of rabies was very high, some important gaps were noted. For instance, more than 90% of the respondents estimated that the maximum incubation period of rabies in humans is less than 40 days. This is consistent with the finding of Agarwal and colleagues [27] who reported that the majority of the communities knew that dog bites could cause death but were not aware of the incubation period of rabies. The incubation period of rabies in humans varies depending on the site of the bite, severity of the wound and amount of virus introduced into the wound and ranges from a few days to several years; in most cases signs develop after one to three months [3,28,29]. That is, the incubation period is not limited to 40 days. Misconceptions about the incubation period in Ethiopia may emanate from misleading information provided by traditional healers. For example, participants stated that they were advised not to cross rivers and travel to health facilities in search of medical care earlier than 40 days post-exposure. According to the healers, if a victim does not develop clinical signs of the disease during this period, the dog initiating the bite was free of rabies.

In this study, the majority (95.6%) of the respondents believed that a person potentially exposed to rabies should seek medical evaluation promptly. This favorable attitude is consistent with recommendations that anyone who has been bitten by an animal, or who otherwise may have been exposed to rabies, should seek medical attention immediately [30]. However, the fact that 75% of the respondents concurrently believed that traditional healers and herbal medicine could cure rabies may suggest that inappropriate assistance may be sought following bite. Indeed, some bite victims in this study stated that they sought medical care because they could not comply with advice provided by a traditional healer. Ayalew indicated that the widespread use of traditional, (supposedly) anti-rabies herbal remedies is common in Ethiopia and noted that some exposed individuals discontinue the vaccination regime to start these herbal remedies [31]. More recently, Deressa and colleagues stated that most fatal human rabies cases recorded at the Ethiopian Health and Nutrition Research Institute were associated with herbal remedies where the majority of human rabies cases were treated by traditional healers [32]. We found that Protestant religion and rural residence were independently associated with a belief that traditional healers could cure rabies, after adjusting for other factors (Table 6). Individuals who use herbal remedies and do not develop disease may believe that they did not contract rabies due the use of the remedy after exposure. However, this may be a false association because not all animal bites will cause rabies.

In this study, only 7% of the respondents indicated that they washed the bite wound with water and soap as first aid, while 43% performed no first aid after being bitten by a suspected rabid animal. Similar low rates of first aid have been reported elsewhere in the Philippines [33] and India [25]. The WHO recommends that wounds be thoroughly flushed with soap and water immediately after a bite injury; povidone iodine or other antiseptic should be applied when available [3]. Early treatment of all bites and scratches is important because the virus can remain within the area of the injury for an indefinite duration of time. It is therefore recommended that first aid be applied even if the person presents long after exposure [30].

In terms of animal management following a bite, 47.7% of respondents replied that they immediately kill suspected rabid dogs that bite humans to prevent further attacks. This is similar to reports from Tanzania where most study participants state they would kill the animal without informing livestock health officers in the areas [24]. Such practices are contrary to recommendations that these animals be quarantined for 10 days to determine whether the exposed human should be vaccinated [34]. In most cases, communities kill dogs as soon as they bite humans but do not submit the animal’s head for rabies diagnosis. In this case, all exposed persons require anti-rabies treatment. In reality, not all bites cause rabies; dogs may bite people due to aggressiveness to strangers, behavioral change during breeding seasons, and to protect newly born puppies. While we do not condone withholding PEP following potential exposure to rabies, administration of PEP in people who were not exposed results in unnecessary costs related to post-exposure treatment and transport to the health center, as well as pain associated with multiple injections with a large needle (the nerve-tissue vaccine (Fermi type) remains the most widely available vaccine in Ethiopia [35]). In addition, it makes it difficult to appreciate the scale of the problem and take appropriate steps to prevent further transmission because dogs are not quarantined and observed for development of clinical signs.

In this study, female sex was independently associated with a higher practices score, while Muslim faith was associated with a lower score. Further investigation revealed that this was due to differences in the way suspect rabid animals were managed, perhaps reflecting dissimilar participation or attitudes towards dogs among these groups. Variation by gender in reported animal management practices after exposure is unlikely to be due to differential recall since males and females were equally likely to report that they did not know the status of the animal (16.7% vs 15.8%, p = 0.883). One study in Ethiopia found that management of dogs was more often the responsibility of women and children and so it is possible that females develop different attitudes towards dogs [16]. Muslim households in this study were more likely to report that the animal was killed. Dogs are considered impure in Islamic tradition [36] and so it is conceivable that people of this faith may be less concerned with dog welfare. Muslim households were less likely to own dogs than other households in this study (39.6% vs 55.0%, p = 0.003). Dog ownership rates among Muslim households were considerably higher in this study compared to a similar study in Addis Ababa (2.6%) [16]. This difference likely reflects the rural focus of the present study; according to Islam, dogs may be used for hunting and guarding crops and livestock [37]. Indeed in Tanzania, where Muslim households were also less likely to own dogs, the effect of religion was not observed when only livestock-owning households were considered [38].

The majority of respondents identified that rabies could be prevented by avoiding dog bites and confining dogs. Despite a good level of knowledge of these preventive measures, these were poorly practiced among dog owners. Only 9% of pet owners tied up their dogs during the whole day. The remaining 91% of dog owners reported that their dogs were untied and free to cohabit with family and forage widely. This is similar to findings from Addis Ababa [16]. Likewise, only 5% of pet owners in the present study vaccinated their dog. Again this is considerably lower than that reported for Addis Ababa, where vaccine is more easily available [16]. Participants also identified eliminating strays or free roaming dogs as a possible control measure. Dog culling is not recommended as a rabies control strategy on its own [39]. However, along with dog vaccination, removal of strays or free roaming dogs may form part of an initial rabies control program in this particular setting where the number of unwanted dogs is considered problematic. Thus, we looked at elimination of free roaming and stray dogs as a short term solution, with awareness creation on responsible dog ownership (confining dogs, vaccination) as a longer term goal.

About 35% of the respondents reported slaughtering food animals suspected of rabies and eating their meat, considering that the meat from such animals has medicinal value. In contrast, 65.4% of the respondents reported killing and avoiding (not eating) the body of animals bitten by suspected rabid animals fearing that rabies is transmitted through an animal’s carcass. Rabies virus is inactivated by heating and therefore eating cooked meat or pasteurized milk is not considered an exposure [34]. However, drinking unpasteurized milk or unprotected cutaneous contact with a carcass (for example, during butchering and dressing) are considered exposures [34]. In another study, about one quarter of respondents in Tanzania claimed they would throw away the carcass of a rabid animal [24]. This habit poses a risk for scavengers, which may feed on dead infected animals. In South Africa, an outbreak of rabies in endangered wild dogs in 2000 was speculated to be associated with feeding on the carcass of a rabid jackal [40]. This suggests the need to create awareness that the carcasses of all animals that have died of rabies should be burned or buried to stop the transmission of rabies to scavengers and other animals.

This study has some limitations. Due to ethical considerations we did not interview minors (defined in this cultural setting as <15 years). Children are a major risk group for rabies [6] and constituted around 40% of the individuals who presented to the health center in this study. We did not evaluate the knowledge, attitudes and practices of these individuals directly but rather interviewed the parent or older sibling who brought them to the health center. In addition, our study focused on people presenting to the health center for treatment following potential exposure. Thus, our findings reflect the knowledge, attitudes and practices of people who have some awareness of medical intervention and may not be generalizable to the entire community. Even so, we found that the view that herbal remedies can cure rabies existed concurrently in the study population. This may suggest that access and availability of medical versus traditional medicines, as well as willingness to comply with the treatment, may be a key determinant of what treatment is sought.

In sum, most of the respondents presenting to JTAHC were familiar with the clinical signs of rabies in animals and humans and with the routes of infection. However, there was a lower level of knowledge of the need to vaccinate and confine dogs. Most of the respondents had good attitude towards the public health risk of rabies but their actual practices, particularly with regard to wound washing with soap and water (first aid) and dog management following possible exposure, was inadequate and does not favor the control of the disease. Gender and religion were found to influence these practices. The fact that the majority of the respondents concurrently believed that herbal remedies cure rabies contributes to unnecessary deaths and underestimation of the burden of rabies, since these cases remain unreported to the health centers. This belief was significantly more common among rural residents and points to the need for specific measures to counter this attitude in these areas.

Campaigns aimed at raising public awareness around responsible pet ownership including regular vaccination and confinement, as well as appropriate first aid and on the use of traditional remedies are needed. Based on the findings of this study, we suggest religious leaders and gender offices should be involved in rabies prevention efforts so that critical gaps can be addressed in culturally- and gender-sensitive ways. For instance, religious leaders can serve as conduits of information about rabies prevention at church and mosque services. Anthropologists and others with expertise in ethnomedicine should also be recruited to rabies prevention efforts, to advise on strategies for communication around the ineffectiveness of herbal remedies. The findings of this study will be shared with the Jimma University Research and Community Based Education Office so that critical gaps identified by this study can be addressed in collaboration with the Jimma Zonal Health Office and other stakeholders.

## Supporting Information

S1 Checklist STROBE checklist. (DOC)

S1 Dataset De-identified dataset used for all analyses presented in this manuscript. (XLSX)
